# Massive acute ischemic stroke after *Bothrops* spp. envenomation in southwestern Colombia: Case report and literature review

**DOI:** 10.7705/biomedica.6114

**Published:** 2022-03-01

**Authors:** Viviana Alexandra Martínez-Villota, Paulo Francisco Mera-Martínez, José Darío Portillo-Miño

**Affiliations:** 1 Facultad de Medicina,Universidad Nacional de Colombia,Bogotá, D.C., Colombia Universidad Nacional de Colombia Universidad Nacional de Colombia Bogotá, D.C. Colombia; 2 Departamento de Neurología,Hospital Universitario Departamental de Nariño, Pasto, Colombia Hospital Universitario Departamental de Nariño Pasto Colombia; 3 Facultad de Ciencias de la Salud,Universidad de Nariño, Pasto, Colombia Universidad de Nariño Facultad de Ciencias de la Salud,Universidad de Nariño Pasto Colombia; 4 Departamento de Emergencias, Hospital Universitario Departamental de Nariño, Pasto, Colombia Hospital Universitario Departamental de Nariño Pasto Colombia; 5 Facultad de Ciencias de la Salud, Grupo de Investigación RIZHOME GROUP II, Fundación Universitaria San Martín, Pasto, Colombia Fundación Universitaria San Martín Pasto Colombia; 6 Grupo de Investigación en Infecciosas y Cáncer,Fundación Hospital San Pedro, Pasto, Colombia Fundación Hospital San Pedro Pasto Colombia

**Keywords:** Stroke, Bothrops, snake bites, snake venoms., accidente cerebrovascular, Bothrops, mordedura de serpiente, venenos de serpiente.

## Abstract

*Bothrops* spp. envenomation and its relationship with ischemic stroke has complex pathogenesis. Local effects such as edema, pain, redness, necrosis, and systemic manifestations like coagulation disorders, thrombosis, renal failure, and hemorrhage have been reported. Hemorrhagic stroke is a common neurological complication but ischemic stroke is poorly understood.

We present here the case of a 50-year-old male with no comorbidities referred from a rural area in southwest Colombia with a *Bothrops* spp. snakebite on the left hand. On admission, the patient presented with a deterioration of consciousness and required mechanical ventilation assistance. The MRI showed multiple ischemic areas in the bilateral frontal- temporal and occipital regions. Two months later, the patient had a favorable resolution, although central paresis in the III and VI cranial nerves and positive Babinski’s sign persisted. As already mentioned, the pathophysiology of ischemic stroke due to snakebite is complex but the procoagulant activity of the venom components, the hypovolemic shock, the endothelial damage, and the thromboinflammation can explain it, and although it rarely occurs, it should be considered as a complication of ophidian accidents caused by *Bothrops* spp.

## Resumen

La mordedura de serpientes *Bothrops* spp. y el ataque cerebrovascular isquémico tienen una patogenia compleja. Se reconocen efectos locales como edema, dolor, enrojecimiento y necrosis, así como manifestaciones sistémicas como trastornos de la coagulación, trombosis, insuficiencia renal y hemorragia, por lo que el accidente cerebrovascular hemorrágico es una complicación neurológica común, pero, en cambio, el accidente cerebrovascular isquémico es poco conocido.

Se presenta el caso de un paciente de 50 años, sin comorbilidades, remitido de una zona rural del suroccidente de Colombia debido a la mordedura de una serpiente *Bothrops* spp. en su mano izquierda. En el momento del ingreso, el paciente presentaba deterioro de la conciencia y requirió asistencia respiratoria mecánica. Mediante resonancia magnética, se observaron múltiples áreas isquémicas bilaterales en la región fronto-temporal y en la occipital. Dos meses después, el paciente había evolucionado favorablemente, pero persistían la paresia en los pares craneales III y VI y el signo de Babinski.

La fisiopatología del accidente cerebrovascular isquémico por mordedura de serpiente es compleja. La actividad procoagulante de los componentes del veneno, el choque hipovolémico, el daño endotelial y la tromboinflamación pueden explicar el accidente cerebrovascular isquémico que, aunque raro, debe considerarse como una complicación del accidente ofídico causado por serpientes *Bothrops* spp.

Snake bites and cerebrovascular attacks (CVA) are complex events whose pathogenesis is not yet fully understood, and CVA is considered one of the most severe complications of snake bites ^1^.

*Bothrops* spp. vipers belong to the Viperidae family, which comprises about 200 species of snakes. Russell's viper (Daboia ruselli), Echis carinatus, Bitis arietans, Crotalus spp., Sistrurus spp., *Bothrops* spp., and Bothropoides spp. are among the most dangerous species for humans. These snakes are mostly found in the Americas, Europe, Africa, and Asia ^2^^-^^4^.

*Bothrops* spp. snakebites produce most of the envenomation in Central and South America. In the Department of Nariño, southwestern Colombia, reports show that *Bothrops* snakes (mainly *Bothrops asper*) have caused 43.6% of ophitic accidents ^5^. *Bothrops* spp. envenomation is known for its complex pathogenesis due to its local effects, such as edema, pain, redness, and necrosis, or its systemic manifestations characterized by coagulation disorders, thrombosis, kidney failure, and hemorrhages ^6^.

Although hemorrhagic stroke is a common neurologic complication of ophidic accidents ^7^^-^^10^, others such as muscle paralysis, neuromuscular junction disorders, ^11^ and ischemic stroke have been described (table 1). The toxic effects of the venom in cerebrovascular events have been described in hemorrhagic stroke; however, it has been argued that the secondary procoagulant effect of various components of the poison, the hypotensive shock, and the vasculitis mediated by the immune system are responsible for ischemic stroke leading to an unfavorable prognosis in most cases ^1^^,^^7^.

**Table 1:** Description of the reported cases of ischemic stroke and *Bothrops* snakebite

No. case	Year and reference	Country	Snakebite	Vascular territory	Laboratory tests on admission	Deficit
1	Numeric, *et al*., Martinica (2002) ^26^	Martinica	*B. caribbaeus*	Right ACA and multiple small foci in cerebellar cortex, right PCA, both MCA	CK: 1212 U/L (26-174); CRP: 147.7 mg/L (0-10); PTT: 29 s (control: 32); fibrinogen 6.33 g/L (2-4); D dimer: positive; PLT 201000	Left hemiplegia and a partial Wernicke’s aphasia
2	Angarita,*et al*., Colombia (2003) ^33^	Colombia	*B. spp.*	Right MCA, left MCA	PT> 2 min; PTT>2 min PLT : 23.500	Ocular deviation to the right, dysarthria, left hemiparesis, bilateral Babinski
3	Merle, *et al*., Martinica (2005) ^32^	Martinica	*B. lanceolatus*	Right PCA	PLT: 52,000 cells/mm3 PT: 14% (70-100%), aPTT: 51 s (normal, 32); fibrinogen <0.5 g/L (2-4 g/L); factor II level 66% (70- 120%); factor V level 17% (70-120%); FDP: 2560 pg/ml (normal <5 g/ml)	Left, lateral, homonymous quadranopsia with macular epargne
4	Thomas, *et al*., Martinica (2006) ^34^.	Martinica	*B. lanceolatus.*	Both PCA	PLT: 57,000; fibrinogen<0,5 (g/l); PT: 34 s; aPTT: 51 sec; FDP: 2560 pg/ml; CRP 1,9 mg/ml	Right hemiparesis and aphasia
5	Thomas, *et al*., Martinica (2006) ^34^.	Martinica	*B. lanceolatus*	Left MCA	PLT: 20000; fibrinogen 2,45 (g/l); PT: 15 s; aPTT: 35 s; FDP: 320 pg/ml; CRP: 25,3 mg/ml	Right hemiparesis and aphasia
6	Thomas, *et al*., Martinica (2006) ^34^.	Martinica	*B. lanceolatus*	Both PCA, left MCA	PLT: 260,000; fibrinogen 2,81 (g/l); PT: 13 s; aPTT: 28 s; CRP: 0 mg/ml	Left hemiparesis and left homonymous hemianopsia.
7	Cañas, *et al*. Colombia	Colombia	*B. atrox*	Basilar artery	PLT: 18,000/mm3, PT>1 min A PTT>1 min; fibrinogen was not detected	Comatose, miotic pupils without reaction to light and generalized hypotonia, lack of response of limbs to painful stimuli, bilateral Babinski
8	Martinez- Colombia Villota *et al*.	Colombia	*Bothrops spp.*	MCA	PT: 44 sec; blood test, normal; liver and renal function, normal; fibrinogen<50 mg/ml	Impaired level of consciousness, agitation, and left hemiparesis

PT: Prothrombin time; PTT: Partial thromboplastin time; acPTT: Activated cephalin time or partial thromboplastin time; PLT: Platelet, CK: Creatine kinase; CRP: C-reactive protein; ACA: Anterior cerebral artery; MCA: Middle cerebral artery; PCA: Posterior cerebral arteries; FDP: Fibrin degradation product

We report here the case of a 50-year-old patient with a massive ischemic stroke caused by *Bothrops* spp. envenomation in a rural area in southwestern Colombia.

## Case report

A 50-year-old male patient with no medical background was referred from a rural area in southwestern Colombia (department of Nariño) to the emergency room of our hospital. He had been bitten by a *Bothrops* spp. snake on the second finger of his left hand. At the primary care hospital, the physical examination showed the progression of the edema to the left arm but no evidence of ulceration, hemorrhage, necrosis, signs of infection, or other findings. The results from the laboratory tests were: Prothrombine time: 44 s, normal complete blood count, and normal kidney function.

**Figure 1:**
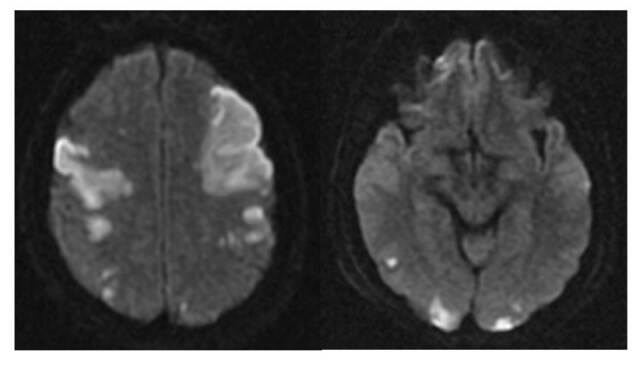
MRI (DWI) showing multiple areas of bilateral fronto-temporal, and occipital ischemia.

Source: Archive of the Department of Diagnostic Images, University Hospital of Nariño, Pasto, Colombia

The patient was treated with nine vials of polyvalent anti-venom serum four hours after his referral. He arrived at our hospital 13 hours after the snakebite; he was hemodynamically unstable, with a deterioration of his level of consciousness, agitation, and left hemiparesis which required management in the intensive care unit (ICU) and mechanical ventilation assistance. The blood test performed showed thrombocytopenia, dysfibrinogenemia with fibrinogen levels below 50 mg/ml, and clotting times without alterations. The patient was evaluated by a neurologist who ordered a brain MRI that revealed multiple ischemic areas in the bilateral frontal-temporal and bilateral occipital regions, as well as pons and cerebellum (figure 1).

Due to the severity of the stroke, he was given an additional anti-venom scheme (each vial contains 10 ml of lyophilized equine polyvalent anti-venom serum which neutralizes at least 25,10, and 5 mg of the venom of *Bothrops asper/atrox, Crotalus*, and *Lachesis* snakes, respectively). The patient did not receive plasma because this would have increased the procoagulant risk and fibrinogen levels gradually in the first 24 hours after the anti-venom treatment. The patient improved as recorded in the follow-up control two months later, but his bilateral paresis on the VI and III cranial nerves persisted with a strength of 2/5 on the left side, as well as left Babinski's sign with a Modified Rankin Scale (mRs) of 4.

We complied with all the ethical regulations for research in humans and no intervention was carried out. We received the informed consent of the patient authorizing the publication of the case and the images.

## Discussion

Snake bites and strokes have been reported in detail in the medical literature. Mosquera, *et al*. ^7^, for example, found a 2.6% prevalence of cerebrovascular complications among 309 victims of snakebites ^7^. Ischemic stroke has been described in other snake species, mainly in *Gloydius brevicaudus, Crotalus durissus terriftous, Hypnale, Echis carinatus,* and Russell’s viper (*Daboia russelii*) ^12^^-^^22^.

A study conducted in Sri Lanka showed that 1.8% of 500 patients bitten by Russell’s viper had an ischemic stroke ^23^. Other studies In Martinique provided a closer panorama of ischemic stroke with pro-coagulant effects provoking up to 22% of thrombotic complications such as pulmonary thromboembolism and myocardial infarction, and 12% of stroke in the patients observed ^24^^-^^26^.

In Colombia, a study of 39 cases found that 12.8% of the patients bitten by a *Bothrops* snake had hemorrhages in the central nervous system, but no ischemic strokes were reported ^27^. In four additional Colombian studies involving around 698 patients, no ischemic strokes were registered ^28^^-^^31^. According to the literature review, and as far as we know, seven cases of ischemic stroke due to *Bothrops* spp. envenomation have been documented ^26^^,^^32^^-^^35^ (table 1) in the world. In our country, only some descriptions are found. Angarita, *et al*., reported a stroke associated with an ophidian accident ^33^, and Cañas, *et al*., recently reported an ischemic stroke in the brainstem caused by a *Bothrops atrox* snakebite ^35^. In this context, the present case is the third one reported in Colombia and the eighth in the world.

The etiopathogenesis of ischemic stroke still has to be fully elucidated ^36^^,^^37^. The most relevant hypothesis currently considered is thromboinflammation activated by the different toxins of the venom. The most representative enzymes in this process are metalloproteases and type-C lectins responsible for stimulating platelet function and pro-inflammatory activity ^38^ causing endothelial injury, stimulation of the immune system, immune-mediated vasculitis, hypercoagulable state, and systemic hypotension ^1^^,^^38^. Other toxins, such as digestive enzymes and complementary factors, produce local and systemic injury. The enzymes causing the most severe reactions are the zinc-dependent metalloproteinases called “hemorrhages” which cause hemorrhage due to hydrolysis of the basal lamina in capillaries ^39^. Similarly, phospholipase A2 is involved in the formation of edema and myotoxicity, and it has anticoagulant effects. Additional studies of snake venom including proteomics have revealed that some venom compounds are acid phospholipases A2, serine proteinases, acid 1-amino oxidases, zinc-dependent metalloproteinase, and specific C-type lectin binding molecules ^40^^-^^42^.

**Figure 2:**
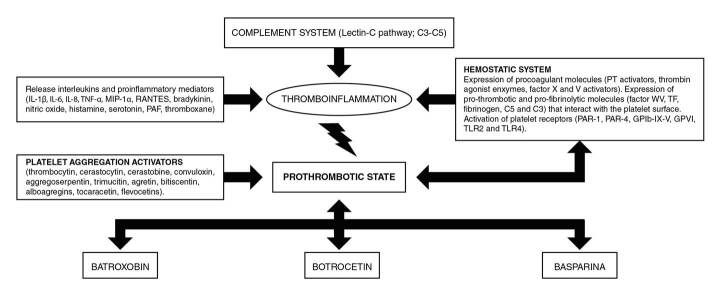
Flow-diagram of probable pathophysiology mechanisms of ischemic stroke in the Bothrops envenomation.

The molecules released in edema and hemorrhages are the metabolites of arachidonic acid (cyclooxygenase and lipoxygenase), bradykinin, histamine, and serotonin ^43^. It has been shown that *Bothrops lanceolatus* snakes are the most frequently associated with systemic thrombotic complications ^26^^,^^44^. It is understood, then, that toxins produce the thrombotic phenomenon through endothelial injury due to the direct action of the venom on the vessels ^6^. Endothelial damage is the result of the synergistic effect of metalloproteinase and phospholipase A2 ^45^. The hyperviscosity produced by the hypovolemic state and hypoperfusion secondary to hypotension contributes to the occlusion of the vessels ^46^. Similarly, some toxins promote the activation of the complement system through the generation of anaphylatoxins hydrolyzing C3 and C5 and activating the three complement pathways, especially the lectin pathway and C1-INH inactivation, which is an important inhibitor of coagulation proteins contributing to inflammation and thrombosis ^47^ (figura 2).

The factor V Leiden has also been considered since it plays a key role in the activation of the anticoagulant and procoagulant pathways and is activated by several toxins and proteases present in the venom of the vipers *Vípera, Naja oxiana, and Bothrops atrox*^48^. In a comparative analysis of the coagulant activity of the venoms of the different *Bothrops* spp. snakes, the venom of *B. erythromelas* showed high levels of factor X and prothrombin activators ^49^. *Bothrops* spp. venoms possess thrombin activating enzymes ^50^. The main molecules associated with the anticoagulant and procoagulant effect of the *Bothrops* snake venom are described in table 2. The cranial nerve involvement could be associated with cerebellar and pons infarction, as well as snakebite- associated ophthalmoplegia that has also been described ^11^.

**Table 2:** Main procoagulant and anticoagulant molecules of the hemostatic system of the *Bothrops* snake

Anticoagulants factors	Procoagulants factors	
	A. Thromboinflammation ^38^	
A. Aspercitin, hemorrhages, metalloproteinases: these substances can cause thrombocytopenia, prolongation PT and PTT, and disseminated intravascular coagulation. They produce hemorrhages of the cerebral parenchymal and subarachnoid ^7^.	Immune system: activation of the function and migration of the leucocytes release of pro-inflammatory mediators (IL-1β, IL-6, IL-8, TNF-α, MIP-1α, NO, histamine, serotonin, PAF, bradykinin, PGE2, TXA2, LTB4, and RANTES) generation of anaphylatoxins (C3 and C5) generation of DAMPS, TF, and vW factor	Hemostatic system: - expression of procoagulant molecules (PT activators, thrombin agonist enzymes, factor X and V activators) - expression of pro-thrombotic and pro-fibrinolytic molecules (vW factor, TF, fibrinogen, C5 and C3) that interact with the platelet surface - activation of platelet receptors (PAR-1, PAR-4, GPIb-IX-V, GPVI, TLR2, and TLR4) activation of platelets by SV-CLRPs of a non-enzymatic pathway by interaction of toxins with the CLEC-2 receptor
B. Activation of c-protein through the activation of the serine proteases	B. Batroxobin: It is a serine protease similar to the thrombin of *Bothrops atrox moojeni* with the ability to coagulate fibrinogen. Thrombin that releases fibrinopeptides a and b from the NH2 terminal domains of fibrinogen Aα and Bβ chains respectively. Batroxabine only releases fibrinopeptide A. Thus, batroxobin binds fibrin in a different way than thrombin, which may contribute to its higher affinity interaction, selective fibrinopeptide A release, and prothrombotic properties ^51^.	
C. Inhibition of factor IX and X	C. Botrocetin: It is a heterodimeric protein snake venom isolated in *Bothrops* jararaca that induces vwf and platelet glycoprotein gpib-dependent platelet agglutination ^52^.	
D. Inhibition of the trombin through the bothrojaracine	D. Basparin a: It was isolated from the venom of *Bothrops asper*; it does not induce local tissue alterations such as hemorrhage, myonecrosis, and edema. It does not induce systemic hemorrhage, thrombocytopenia, or prolongation of the bleeding time following intravenous administration. At low doses, the only observed effect induced by basparin a when injected intravenously or intramuscularly into mice is defibrinogenation. At higher doses, intravenous administration resulted in sudden death due to numerous occlusive thrombi in the pulmonary vessels ^53^.	
E. Inhibition of the complexprothrombinase: action through the phospholipase A2	E. Aspercitin, hemorrhages, and metalloproteinases: The cause of injury to the blood vessels wall produce cerebral infarct ^7^.	
F. Fibrinolytic activity: adamalysin, fibrolase, atroxase, lebetase	F. Complement system: Activation of the 3 complement pathways, especially the lectin-C pathway through the hydrolysis of peptides C3, C4, and C5. The venom also acts directly on the C5a fragment for the generation of C5a convertase. The C1 inhibitor is a serine protease that regulates the complement cascade (inhibits C1r, C1s, and MASPs), and the coagulation cascade acting on fibrinolytic proteins (kallikrein, FXIIa, FXIa, and plasmin) ^46^	
G. Platelet aggregation inhibitors: α-fibrinogenase, phospholipase A2, 5'-nucleotidases, jarahagin, catrocollastatin, crovidisin, disintegrins, cerastatin, barbourin, albolabrin, cistrin, flavoviridine, elegantin, rhodostatin, tigramine ^53^	G. Platelet aggregation activators: Thrombocytin, cerastocytin, cerastobine, convuloxin, aggregoserpentin, trimucitin, agretin, bitiscentin, alboagregins, tocaracetin, flavocetins ^54^	

PGE2: Prostaglandins; TXA2: Thromboxane; LTB4: Leukotrienes; C3 and C5: Complement fragments; DAMPs: Molecular patterns associated with damage; TF: Tissue factor; vW: von Willebrand factor; PAR-1, PAR-4: Protease activating receptors; GPIb-IX-V: GPIb-IX-V glycoprotein complex; GPIV: Glycoprotein VI;TLR2 and TLR4:Tolllike receptors; CLEC-2: C-type lectin receptor

## Conclusion

The pathogenesis of the *Bothrops* envenomation and the thrombotic phenomenon is complex. There are numerous theories that attempt to explain their mechanism but further research is required for their full understanding. The thromboinflammation theory explains some of the local and systemic effects, such as thrombocytopenia, prolonged clotting times, disseminated intravascular coagulation, and thrombotic events. Procoagulant activity, hypovolemic shock, and endothelial injury have been considered as factors that promote ischemic stroke. In the case we report, despite an early initiation of the treatment, a catastrophic ischemic stroke occurred resulting in considerable disability of the patient. It is imperative to understand this phenomenon as a severe neurological complication in American countries as Colombia where *Bothrops* snakes are widely distributed and ophidian accidents are a frequent reason for consultation in the emergency room.
